# Engineering a branching sucrase for flavonoid glucoside diversification

**DOI:** 10.1038/s41598-018-33394-y

**Published:** 2018-10-11

**Authors:** Yannick Malbert, Claire Moulis, Yoann Brison, Sandrine Morel, Isabelle André, Magali Remaud-Simeon

**Affiliations:** Laboratoire d’Ingénierie des Systèmes Biologiques et Procédés, LISBP, Université de Toulouse, CNRS, INRA, INSA, Toulouse, France. 135, avenue de Rangueil, F-31077, Toulouse, cedex 04 France

## Abstract

Enzymatic glycosylation of flavonoids is an efficient mean to protect aglycons against degradation while enhancing their solubility, life time and, by extension, their bioavailability which is critical for most of their applications in health care. To generate a valuable enzymatic platform for flavonoid glucosylation, an α-1,2 branching sucrase belonging to the family 70 of glycoside-hydrolases was selected as template and subsequently engineered. Two libraries of variants targeting pair-wise mutations inferred by molecular docking simulations were generated and screened for quercetin glucosylation using sucrose as a glucosyl donor. Only a limited number of variants (22) were retained on the basis of quercetin conversion and product profile. Their acceptor promiscuity towards five other flavonoids was subsequently assessed, and the automated screening effort revealed variants showing remarkable ability for luteolin, morin and naringenin glucosylation with conversion ranging from 30% to 90%. Notably, naringenin and morin, *a priori* considered as recalcitrant compounds to glucosylation using this α-transglucosylases, could also be modified. The approach reveals the potential of small platforms of engineered GH70 α-transglucosylases and opens up the diversity of flavonoid glucosides to molecular structures inaccessible yet.

## Introduction

Flavonoids are natural pigments constituting a class of widespread phenolic compounds in plants. These secondary metabolites have different biological functions depending on their structures and can play critical roles, for instance as attractants of pollinators, protective agents against UV radiation or oxidation, repellents of phytopathogens to name just a few^1,2^. Identified as important bioactive components of many traditional medicine formula, numerous reports indicate correlations between flavonoid-rich diets and a lower prevalence of chronic diseases, such as cardiovascular or neurodegenerative diseases, type II diabetes, and possibly cancers^3,4^. These findings undoubtedly stimulate research on structure-bioactivity relationships of flavonoids for therapeutic applications^5^. Besides their natural occurrence in food diets, flavonoids are used in food industry as dietary supplements, color additives, and/or antioxidants^6,7^. They are also very appealing for the cosmetic industry, where they are mostly used for their antioxidant, UV filter, skin blood vessels protection and/or soothing actions^8^.

However, their weak solubility in either aqueous or hydrophobic environments, sometimes accompanied by a poor stability, renders their formulation uneasy, limits their bioavailability and, consequently, their expected biological effects. In plants, flavonoids are often found in the form of C- or O- glycosides. Glycosylation is obviously an efficient way to generate derivatives, which can be more soluble and sometimes more stable than their aglycon counterparts^9^. These glyco-derivatives are sought-after molecules but they are often naturally produced in low amounts and their extraction remains quite tedious. As an alternative, chemical glycosylation can be envisioned but chemical processes are also tedious due to the reactivity of the multiple hydroxyl groups, requiring multiple protection and deprotection steps. Altogether, these limitations place enzymatic glycosylation in a favorable position to enlarge the diversity of flavonoid glycosides^10^. Leloir glucosyltransferases are the natural enzymes involved in flavonoid glycosylation *in vivo*, but they use nucleotide-activated sugars as donor substrates, what disadvantages their usage for *in vitro* transformation. From this point of view, transglycosylases are more interesting enzymes as they use more available substrates. This is the case of the sucrose active enzymes from Glycoside-Hydrolase (GH) families 13, 70, 32 and 68 in the CAZy classification^11^, which have received much attention in the two last decades^12^. If examples of flavonoid fructosylation remain scarce, flavonoid glucosylation using GH13 and GH70 enzymes has been described since the mid-nineties by Nakahara *et al*.^13^ and Meulenbeld *et al*.^14^, their work focusing on catechin glucosylation. Then, various flavonoid glucosides including luteolin, phloretin, epicatechin, myricetin, quercetin, ampelopsin, astragalin, baicalein, taxifolin, tri-hydroxyisoflavone have been obtained using native or recombinant enzymes in pure form, crude cell extracts or resting cells^10,15^. In addition, protein engineering enabled the generation of α-transglucosylases that outperform parental wild-type enzymes. A mutant library of *N. polysaccharea* amylosucrase (*Np*AS) was recently screened, leading to the identification of improved mutants producing luteolin mono-, di- and tri-glucosides with considerably enhanced solubility^16^.

To date, the α-transglucosylases from GH13 and GH70 families that were tested for flavonoid glucosylation are essentially glucansucrases (GS) that naturally use largely available sucrose substrate to catalyze the formation of high molar mass homopolysaccharides of glucosyl units composed of α-1,2-, α-1,3-, α-1,4- and α-1,6- osidic linkages depending on the enzyme specificity^15,17^. In GH70 family, a subgroup of α-transglucosylases, named branching sucrases (BrS) due to their ability to produce highly branched dextrans from sucrose substrate and linear α-1,6-linked dextran acceptor, was described^18,19^. Like all the sucrose-active GH70 enzymes, they follow an α-retaining mechanism. The first characterized branching sucrase is a truncated form of the bifunctional glucansucrase DSR-E from *Leuconostoc mesenteroides* B-1299 (reclassified as *L. citreum* B-1299). This enzyme named Δ_N123_-GBD-CD2 (Δ_N123_-glucan-binding domain-catalytic domain 2) was shown to specifically catalyze the formation of α-1,2 linkages. The three-dimensional structure of this enzyme further enabled the identification of the catalytic residues predicted to be involved in the formation of the covalent β-glucosyl enzyme intermediate and the location of the subsites −1 and +1 accommodating sucrose substrate^20^. Notably, this enzyme was never tested before for flavonoid glucosylation although its exposed active site could favor glucosylation of large molecules and its unique linkage specificity could help to access a novel diversity of glucosylation patterns compared to glucansucrases.

In this work, the focus was placed on the semi-rational design of the α-(1→2) branching sucrase, Δ_N123_-GBD-CD2, with the objective of generating a platform of diversified mutants of interest for flavonoid glucosylation. To this end, molecular modeling studies were first carried out to identify amino acid residues in the catalytic pocket, which could be changed to enhance recognition of quercetin, a flavonoid naturally recognized by the wild-type enzyme. Two libraries of ~1,500 mutants each were first generated and screened using a solid pH-based assay enabling the discrimination of sucrose-active mutants. The active clones were then further tested for quercetin glucosylation, leading to the constitution of a small set of nearly neutral mutants both active on sucrose and quercetin. This neutral platform of variants was subsequently assayed for the glucosylation of six distinct flavonoids, namely quercetin, luteolin, morin, naringenin, apigenin and chrysin. Of them, morin, naringenin, apigenin and chrysin had never been tested before with GH70 enzymes. Our results demonstrate the versatility of the platform to generate diversified and novel patterns of flavonoid glucosides.

## Results and Discussion

### Structurally-guided libraries of α-(1→2) branching sucrases

**Figure 1:**
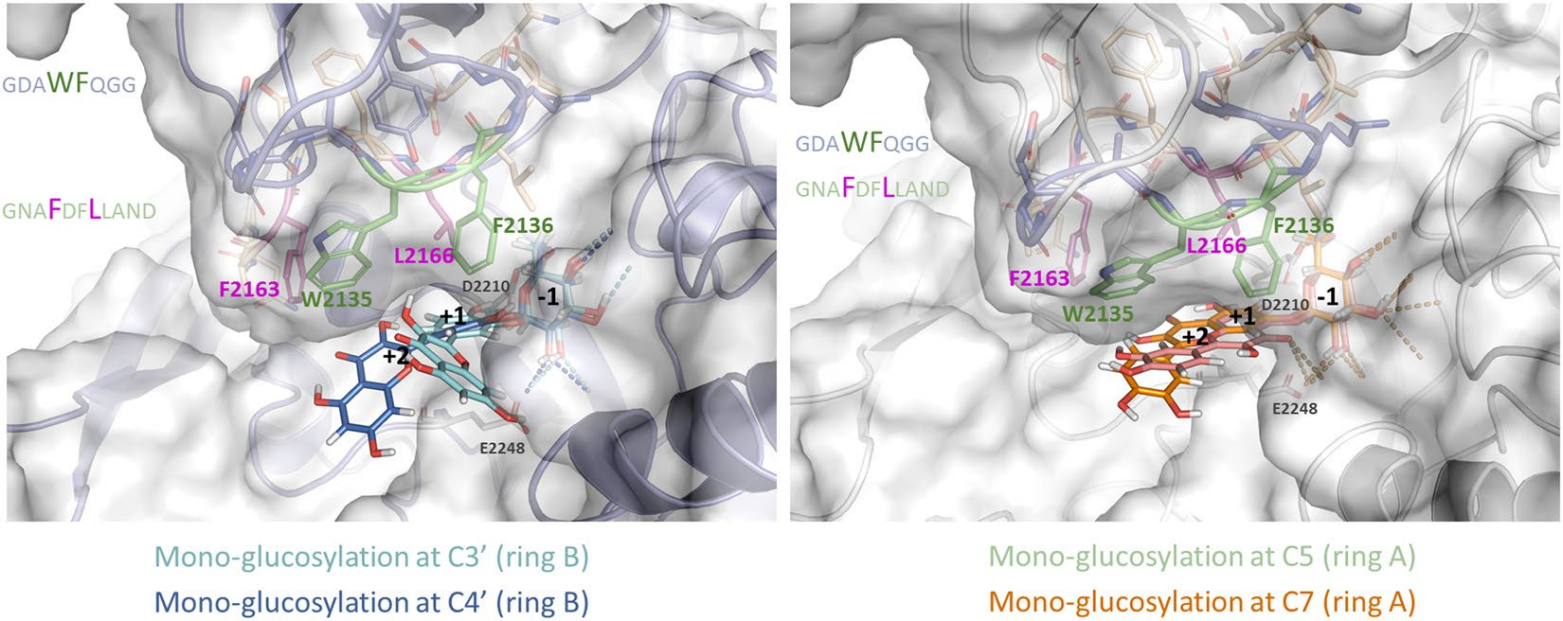
View of the α-(1→2) branching sucrase active site pocket showing the 4 amino acid mutagenesis targets of each library (W2135 and F2136 in light green and F2163 and L2166 in magenta) and the adjacent ones (colored respectively in blue and light pink). Quercetin mono-glucosides (A) glucosylation at C3′ and C4′ of ring A; B: glucosylation at C5 and C7 of ring A) are docked in the active site and represented in sticks. The nucleophile (D2210) and the acid/base (E2248) catalysts are shown in black for reference.

To target putative mutations of interest for improving flavonoid recognition and glucosylation, we constructed several models of the α-(1→2) branching sucrase in complex with all the quercetin monoglucosides that could be obtained from quercetin glucosylation at positions C3′ or C4′ of ring B and at positions C5 or C7 of ring A (Fig. 1). Glucosylation at position 3 of ring C from quercetin was left out due to major steric hindrance. From a detailed comparative analysis of the various docking complexes combined with sequence analysis of amino acid conservation within GH70 family, four amino acids (ie W2135, F2136, F2163 and L2166) located in the motif V, characteristic of GH family 70, upstream the helix α3 of the α-(1→2) branching sucrase, were selected. These four residues from subsites +1, +2, and +3 are not directly involved in catalytic mechanism or sucrose binding. In contrast, they were found systematically in interactions with the mono-glucosylated quercetin molecules docked in the active site, although the nature of the interaction varied depending on the considered molecular structure. The idea was then to authorize random substitutions of these key selected positions by amino acids that could enable the formation of either hydrogen bonds or van der Waals interactions with the quercetin moiety to increase the affinity toward the non-natural acceptor, or to unclutter the active site in order to make enough space for accommodation of the bulky quercetin conjugated system.

Previously, we demonstrated the benefits of pairwise combinations to create or enhance specificity toward non-natural acceptors of GH13 amylosucrases^21^. Given the location of the amino acids, herein targeted, we opted for a similar strategy based on the construction of two sub-libraries, the first one focusing on positions 2135 and 2136, and the second one on positions 2163 and 2166. Pairwise combinations of polar, charged and hydrophobic residues were authorized using a saturation mutagenesis approach and NDT degenerated codons to exclude some amino acids but conversely allow a more extensive exploration of the generated diversity while reducing screening efforts^22^.

### Library screening

**Figure 2:**
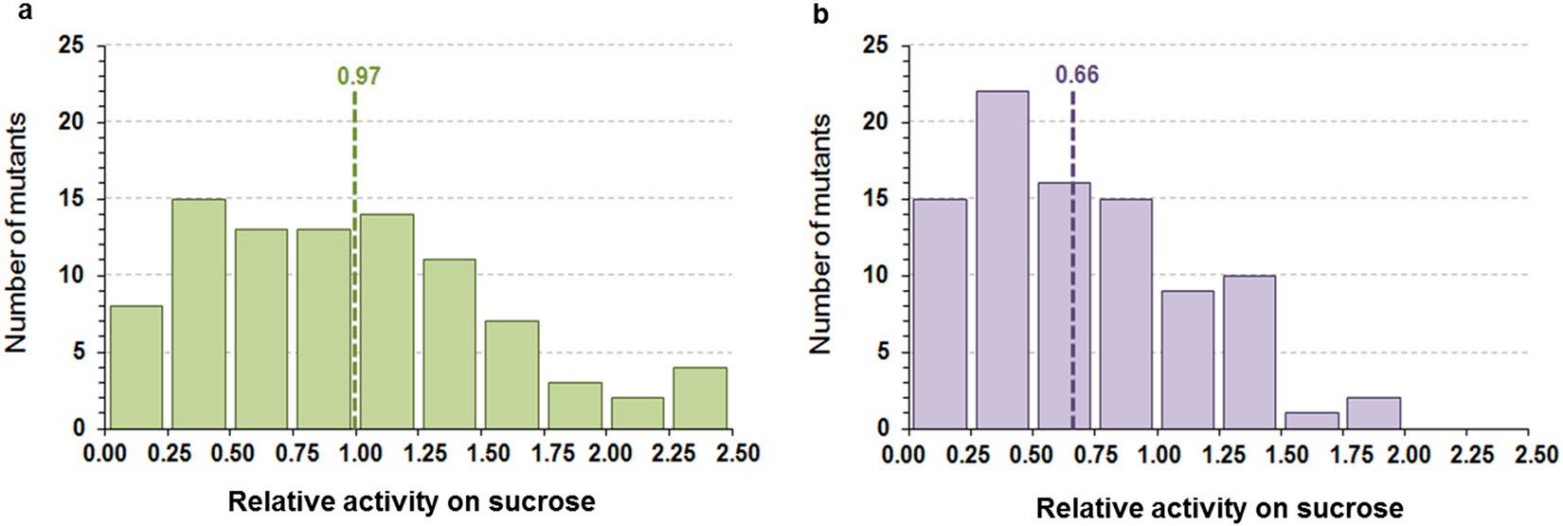
Distribution of the mutant relative activity on sucrose. (**a**) Library 2135–2136 (green) and (**b**) library 2163–2166 (purple). Sucrose cleavage activity was determined by measurement of reducing sugars obtained from sucrose cleavage and expressed as relative activity to the wild-type. Green and purple doted lines represent the average relative activity of library 2135–2136 and 2163–2166, respectively.

A pH based screening assay was set up to distinguish clones having the capability of cleaving sucrose, thus revealing the presence of an active branching sucrase. The assay was validated on clones expressing either the wild-type enzyme or an inactive mutant (E2248Q) and subsequently used to screen 1,500 recombinant clones from each library. This ensured a high coverage of the variant space as to reach a 95% coverage 430 clones should have been screened. We found that 19% and 16% of the clones were still active on sucrose, from 2135–2136 and 2163–2166 libraries, respectively. For each library, 92 active clones were picked at random and their sucrose cleavage activity was confirmed by determination of the reducing sugars formed from sucrose in liquid medium. In library 2163–2166, the average relative activity of the 92 active mutants was 0.66, indicating that the mutations were globally detrimental to sucrase activity (Fig. 2). This may be mostly attributed to mutations at position 2166 of subsite +1. Although L2166 is not in direct interaction with sucrose in the model of the complex α-(1→2) branching sucrase:sucrose, this residue may play a role in the structuring of subsite +1, and consequently in the productive accommodation of the fructosyl moiety of sucrose. In contrast, most of the mutants in library W2135-F2136 were not affected for sucrase activity, suggesting that these two positions located in +2 and +3 subsites are more tolerant to mutations and do not significantly affect sucrose recognition and cleavage. Moreover, six clones presented a two-fold increase of their sucrase activity, when compared to the wild-type enzyme. These 184 mutants were further tested for their capacity to glucosylate quercetin.

Acceptor reactions were performed in the presence of 5 mM quercetin and 292 mM sucrose. The acceptor reaction products were analyzed by LC-MS to examine the product profile and determine the flavonoid conversion. In library 2163–2166, 41% of the clones showed an improved conversion of quercetin compared to the wild-type enzyme for which 60% ( ± 7%) conversion was obtained. Approximately 80% of the mutants from library 2135–2136 converted more than 70% of quercetin. Of note, quercetin was reported to be poorly glucosylated by other glucansucrases. Indeed, previous studies indicated that only 23% of 2 mM quercetin were glucosylated with a glucansucrase from *L. citreum* B-1299^23^ and 4% of 9 mM with the alternansucrase from *L. mesenteroides* NRRL B-23192 (reclassified as *L. citreum* NRRL B-23192)^24^. The conversion values we obtained are much higher, indicating that the wild-type α-(1→2) branching sucrase accommodates more easily quercetin in the acceptor subsites, hence confirming that this template was a good choice to pursue engineering effort.

### Mutant sub-selection

**Figure 3:**
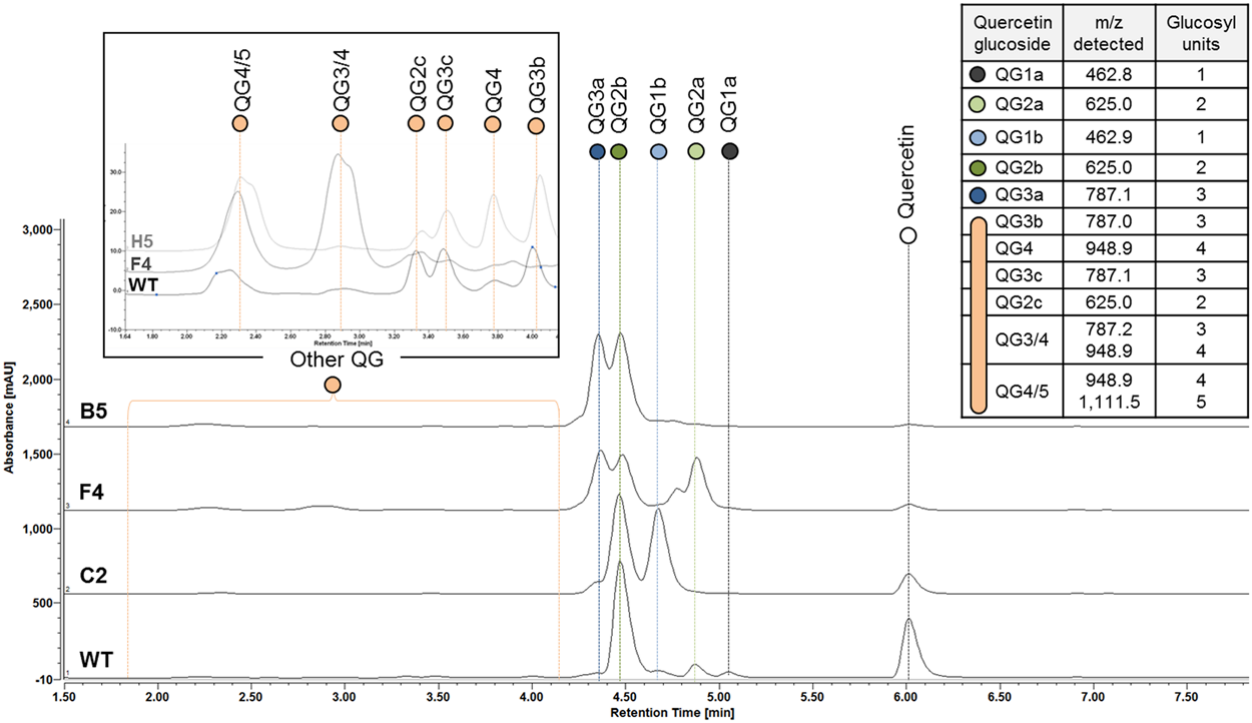
Representatives of the quercetin glucoside profiles. Selection of three typical glucoside profiles identified from LC/MS analysis of the products obtained from quercetin glucosylation using wild-type and branching sucrase mutants (C2, F4, B5 and H5) from library 2135–2136. The mass spectrometry results are also given for each product along with the determination of the number of glucosyl units. Quercetin glucosides are named as QGXn, where Q means Quercetin, G means Glucoside, X is the number of glucosyl units detected by mass spectrometry and n a letter to differentiate the products harboring the same number of glucosyl units.

The LC-MS profiles of the acceptor reaction products obtained from quercetin with the wild-type α-(1→2) branching sucrase and its mutants were analyzed and compared (Figs 3 and S2).

With the wild-type enzyme, two mono-glucosides (QG1a and QG1b) three di-glucosides (QG2a, QG2b and QG2c), three tri-glucosides (QG3a, QG3b, and QG3c) and products showing higher DPs (QG4 and QG5) were identified. The most abundant product is a quercetin di-glucoside (QG2b). The presence of two series of mono-glucosides demonstrates that the wild-type enzyme can graft glucosyl units at least on two different hydroxyl groups of the flavonoid. Quercetin glucosides harboring up to five glucosyl units were also detected.

A total of 16 mutants of library 2135–2136 were retained, including the mutants showing the highest conversion of quercetin (above 90%) and those for which glucosylation profiles diverged from the wild-type one (Table 1 and Supplementary Fig. S2). Among them, mutant F4 produced the highest amount of QG2a (4 times more than the wild-type enzyme). It was also the best producer of QG3/4 and QG4/5. Mutants B5, C12, B9, H1 and C1 showed an improved production of QG3a that represents 40 to 50% of the glucosylated products versus only 2% of the products synthesized by the wild-type enzyme. Mutant D10 was also retained for its ability to synthesize higher amounts of mono-glucoside QG1b compared to the wild-type enzyme. Only 11 mutants were selected from the second library. Eight of them converted quercetin with conversion values ranging from 75% to 89% and the three others (B’5, C’5 and E’10) catalyzed mainly the formation of mono-glucoside QG1b. The 27 retained mutants were sequenced (Table 1).

**Table 1:** Main features of the 22 selected α-(1→2) branching sucrase mutants.

Name	AA at position				Relative activity on sucrose	Quercetin conversion rate (%)
	2135	2136	2163	2166		
WT	W	F	F	L	1.0 ± 0.2^a^	62 ± 7^a^
A10	**V**	F	F	L	2.0	97
B5	**C**	**I**	F	L	0.3	99
C12	**S**	**L**	F	L	0.8	97
C1	**I**	**C**	F	L	0.5	97
C2	**N**	**Y**	F	L	0.4	91
C8	**N**	F	F	L	0.3	91
D10	**I**	**Y**	F	L	1.7	75
E2	**C**	F	F	L	0.5	99
E12	**L**	F	F	L	0.4	98
F3	**N**	**H**	F	L	0.3	91
F4	**L**	**L**	F	L	0.4	97
G1	**F**	**I**	F	L	0.5	98
H1	**C**	N	F	L	0.5	97
H5	**F**	F	F	L	2.0	91
H10	**G**	F	F	L	0.4	98
B’2	W	F	**G**	L	0.5	78
B’5	W	F	F	**I**	0.3	68
C’5	W	F	**H**	L	0.4	76
E’10	W	F	**G**	**I**	0.4	65
F’9^b^	W	F	**L**	L	0.7	89
G’5	W	F	**L**	L	0.9	87
H’5^c^	W	F	**I**	**I**	1.0	70

Mutated residues are in bold. Relative activity to the wild-type (WT) was determined from reducing sugars obtained from sucrose only, measured via DNS assay. Quercetin conversion was calculated from HPLC analysis.

^a^Standard deviations calculated from 3 replicates.

^b^Mutant F′9 exhibit the unexpected additional mutation A2162E.

^c^Mutant H’5 exhibit the unexpected additional mutation D2164E.

In library W2135-F2136, only mutants B9 and C12 were redundant. In addition, 66% of the mutants showed a mutation at position 2136, whereas all of them were mutated at position 2135, indicating that this position is more tolerant to changes and that substitution of the tryptophan bulky side-chain helps to better accommodate quercetin and reach a conversion higher than 90%. In library 2163–2166, mutants D’3, G’2, G’8, and G’10, showing a product profile and conversion close to that of the wild-type, were false positive. In addition, residue L2166 was found mutated only in three variants and replaced by a structurally close isoleucine residue, emphasizing that leucine may be the best amino-acid residue at this position of the +1 subsite for both the sucrose and quercetin accommodation. In addition, two efficient mutants F’9 and H’5 exhibited unexpected mutations (A2162E and D2164E, respectively) not targeted in our constructions.

Finally, a platform of 22 different mutants that could be considered as neutral mutants with regard to sucrose and quercetin conversion were retained and tested for their ability to glucosylate different other flavonoid structures.

### Investigation on the acceptor promiscuity of the mutant platform

**Figure 4:**
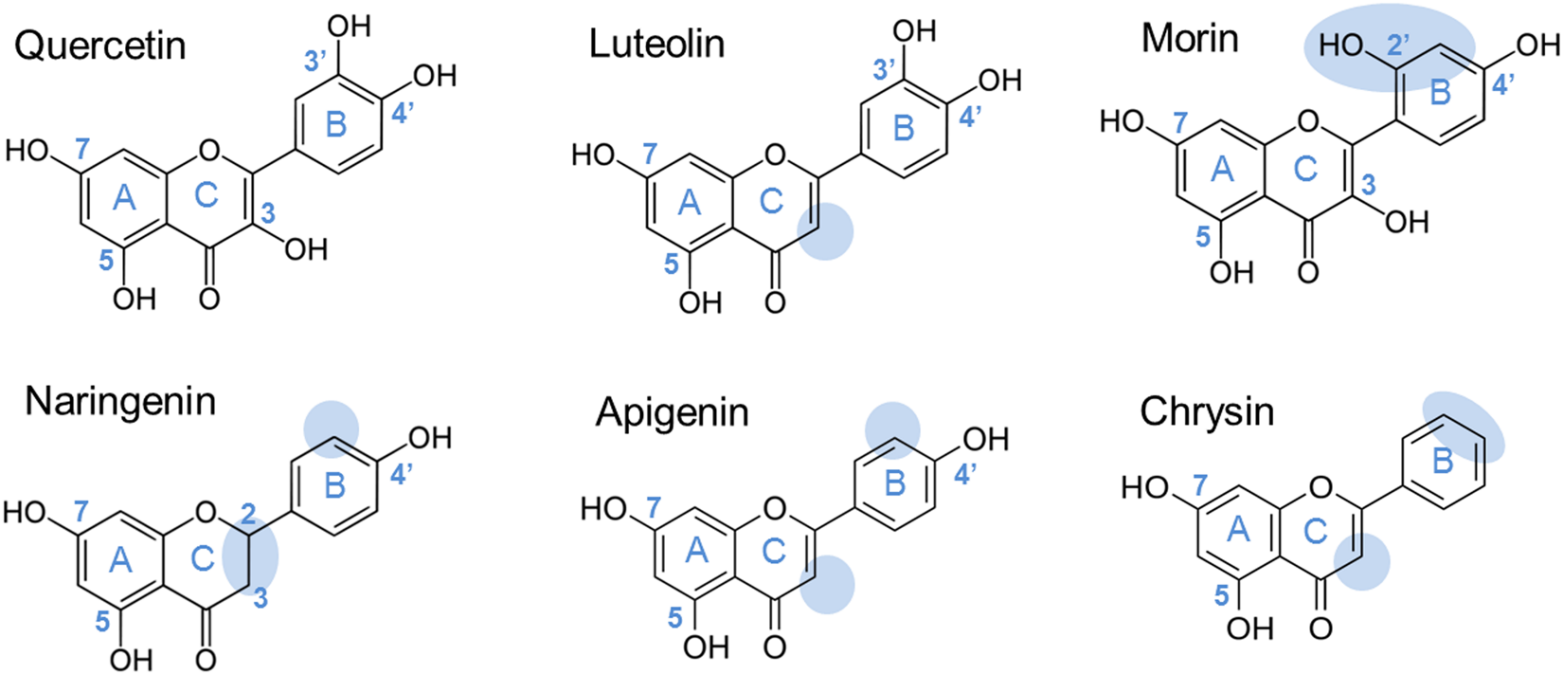
Flavonoids tested as acceptor for the set of 22 selected mutants. Main Structural differences, compared to quercetin, are highlighted with blue circles.

The promiscuity of our mutant platform towards six different flavonoids including quercetin, luteolin, apigenin, morin, chrysin and naringenin was assessed by HPLC-MS analysis of acceptor reactions performed in the presence of 292 mM sucrose and 5 mM flavonoids (Fig. 4).

**Figure 5:**
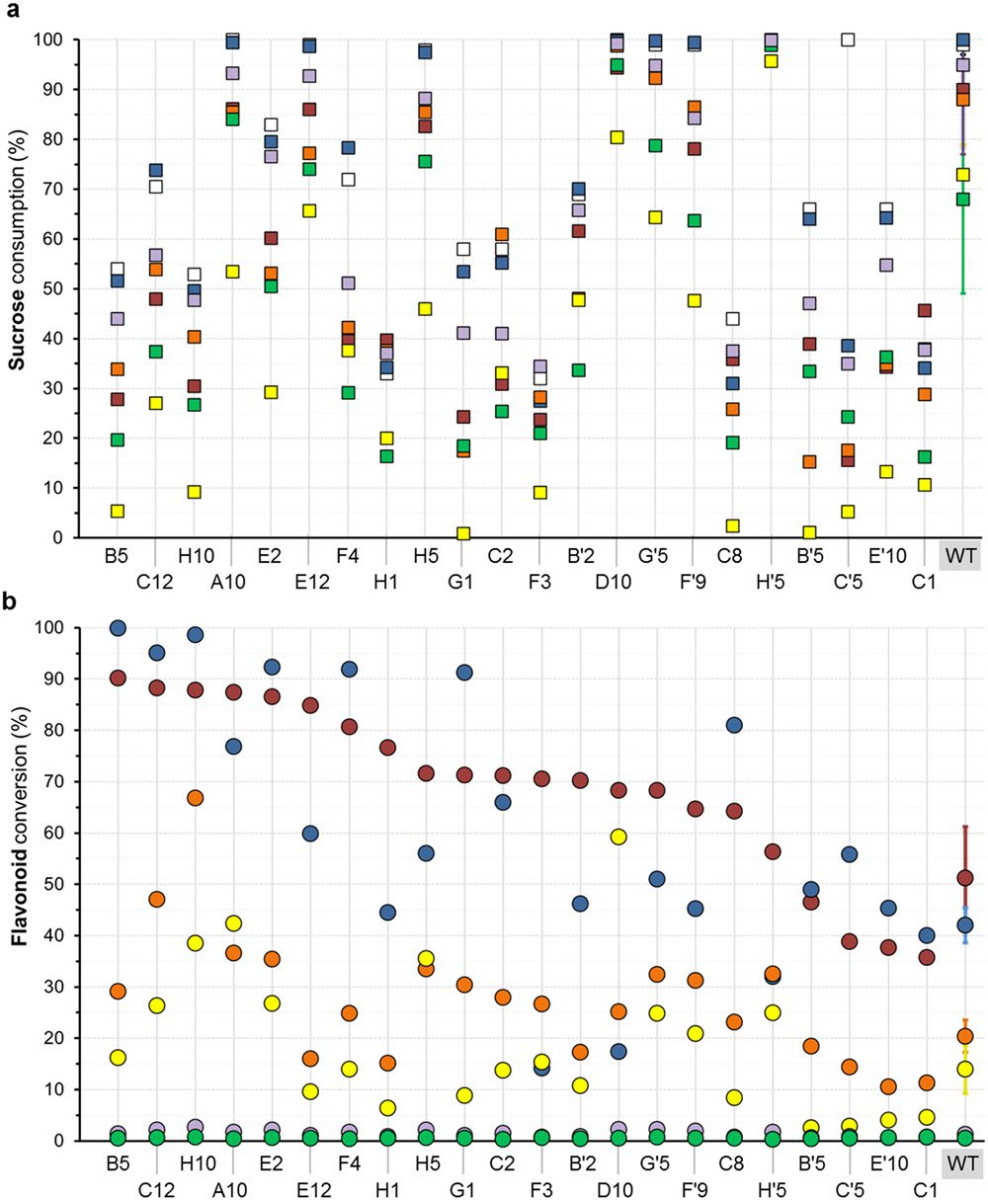
Sucrose and flavonoid conversion obtained with WT and the 22 mutants. Acceptor reactions were conducted with 5 mM of flavonoid and 292 mM of sucrose. Standard deviations given for the WT were calculated on 20 replicates. (**a**) Sucrose consumption in the presence or absence of flavonoids are represented as colored square: □ no flavonoid,  quercetin;  luteolin;  morin;  naringenin;  apigenin;  chrysin (**b**) Flavonoid conversion degrees are represented as colored spots for the wild-type enzyme (WT) and each mutant:  quercetin;  luteolin;  morin;  naringenin;  apigenin;  chrysin.

Like quercetin, morin also belongs to the flavonol subclass but possesses two non-vicinal hydroxyl groups in ring B at positions 2′ and 4′. Compared to quercetin, luteolin is a flavone characterized by the absence of hydroxyl group at position 3 of ring C. Apigenin and chrysin also belong to the flavone subclass like luteolin but chrysin is not substituted with hydroxyl groups on ring B and apigenin is only substituted with one hydroxyl group at position 4′ of ring B. Finally, naringenin, a flavanone, exhibits a less planar structure than the other compounds due to the lack of C2-C3 double bond in C ring. Sucrose consumption and flavonoid conversion obtained after 24 hour reaction are reported in Fig. 5a,b, respectively.

Half of the mutants consumed between 32 and 80% of sucrose and the others more than 80% in the absence of flavonoid acceptors. For nearly all reactions, sucrose conversion decreased upon addition of the flavonoid, whatever their structure was (Fig. 5a). The inhibition effect was moderate (below 30% based on sucrose consumption) for most of the flavonoids. With a total sucrose conversion dropping down to 39% in the presence of the six different flavonoids, mutant C’5 (F2163H) was the most sensitive mutant to flavonoids.

Notably, we did not find any correlation between the type of flavonoid and sucrose conversion (Fig. 5). For illustration, mutant H10 (W2135G) is inhibited up to 83% for sucrose consumption but stands as one of the best mutant for naringenin conversion (38%). Similar levels of naringenin conversion were obtained with mutants A10 (W2135V) and H5 (W2135F) but these two enzymes were however considerably less inhibited than mutant H10 for sucrose conversion. The absence of correlation highlights the subtle differences existing between the different flavonoid-mutant couples and may reflect different binding modes (productive or non-productive) of the flavonoid depending on the mutant.

### Quercetin and luteolin glucosylation

Quercetin and luteolin were more efficiently glucosylated than any other tested flavonoids with all the mutants, excepting mutant D10 (W2135I-F2136Y), which converted nearly three-times more naringenin than luteolin (59% and 17%, conversion, respectively). In contrast to the wild-type enzyme, 9 mutants (B5, C12, E2, F4, G1, C8, C’5, E’10, and C1) better converted luteolin than quercetin, six of them showing remarkable conversion above 90% from 5 mM luteolin.

**Figure 6:**
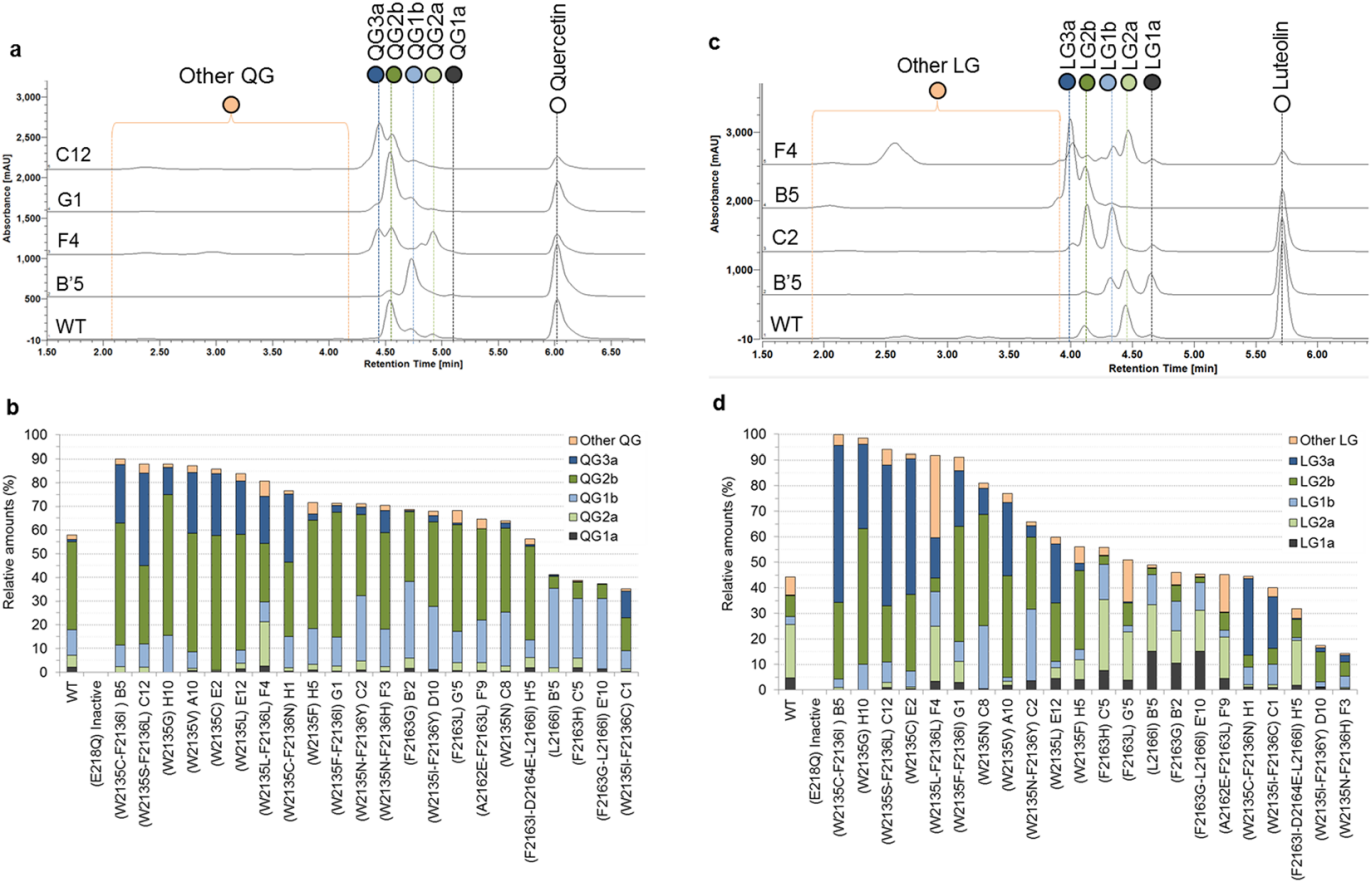
Quercetin and luteolin glucosylation profiles obtained with the 22 selected mutants. HPLC profiles of the best mutants for the production of each glucoside are reported for (**a**) quercetin and (**c**) luteolin. The products are named as XGxn, where X is Q for quercetin and L for luteolin; G means Glucoside; x is the number of glucosyl units detected by mass spectrometry and n a letter to differentiate the products harboring the same number of glucosyl units. A table of MS data is also supplied as Supplementary Table S3. The relative amount of the glucosylation products obtained with the 23 selected mutants are given for (**b**) quercetin and (**d**) luteolin. Mutants are ranked relatively to the highest conversion rates. The products of low retention times produced in lower amounts are grouped under the denomination “Other QG” and “Other LG” are detailed in the Supplementary Table S3.

The results obtained with quercetin were consistent with those observed in the preliminary screening. Four glucosylation profiles of quercetin can be distinguished (Fig. 6a,b). One group produces a majority of mono-glucoside QG1b and di-glucoside QG2b with a profile equivalent to the wild-type one. A second group (mutant **B’5**, E10, C’5) catalyzes mainly the formation of mono-glucoside QG1b (up to 4-fold increase compared to the wild-type). Mutant **F4** (W2135L-F2136L) significantly synthesizes quercetin di-glucoside QG2a, representing 19% of the converted quercetin, and equivalent amounts of QG2b and QG3a. The last type of profiles is illustrated by that of mutant **C12**, which produces up to 40% of quercetin tri-glucoside QG3a, a compound nearly not synthesized by the wild-type enzyme. The 4 best mutants of this category are all members of the library 2135–2136 and possess a cysteine or a serine replacing the tryptophan at position 2135. Finally, traces of mono-glucoside QG1a were also observed on some spectra indicating that glucosylation can principally occur on two different hydroxyl groups of the flavonoid.

We also distinguish 4 different LC/MS profiles resulting from luteolin glucosylation (Fig. 6c,d). The first profile is represented by **B’5** mutant profile. Compared to other variants that consume equivalent amount of sucrose, B’5 mainly produces LG1a and LG1b mono-glucosides as well as LG2a, indicating that the introduced mutations may limit polyglucosylation. In the profile corresponding to mutant C2 (and C8), LG1b and LG2b glucosides are mainly produced whereas in the third group, five mutants (**B5**, C12, E2, H1, and C1) mainly synthesize tri-glucoside LG3a. The same mutants also mainly produce QG3a and exhibit W2135C or W2135S mutations, which clearly favor the tri-glucosylation of these flavonoids. Finally, three mutants (**F4**, G’5 and F’9) are characterized by their ability to produce glucosides of higher degree of polymerization. Notably, mono-glucosides LG1a and LG1b are detected from all the profiles. The structure of LG1b is likely to be a luteolin-4’*-O-*mono-glucoside. Indeed, LG1b co-elutes with the luteolin-4′*-O-*mono-glucoside, a compound synthesized with mutants of *Neisseria polysaccharea* amylosucrase, purified and characterized by NMR analysis^16^. The structure of LG1a is more elusive. However, due to the reactivity of the vicinal hydroxyl groups, we may suspect glucosylation on the 3′O position of ring B. The identification of four di-glucosides indicates that glucosylation may also occur on ring A and/or that di-glucosylation results from transfer on the two mono-glucosides initially formed, but with different types of osidic linkages. Only the isolation and structural characterization of the various compounds, not undertaken within our study, could provide the answer. However, many of the mutants generated are far more efficient than the mutants of *NpAS* previously reported to convert 60% of 5 mM luteolin in similar screening conditions^16^.This highlights the interest of our approach to generate diversity and improve glucosylation yields.

### Morin and Naringenin

**Figure 7:**
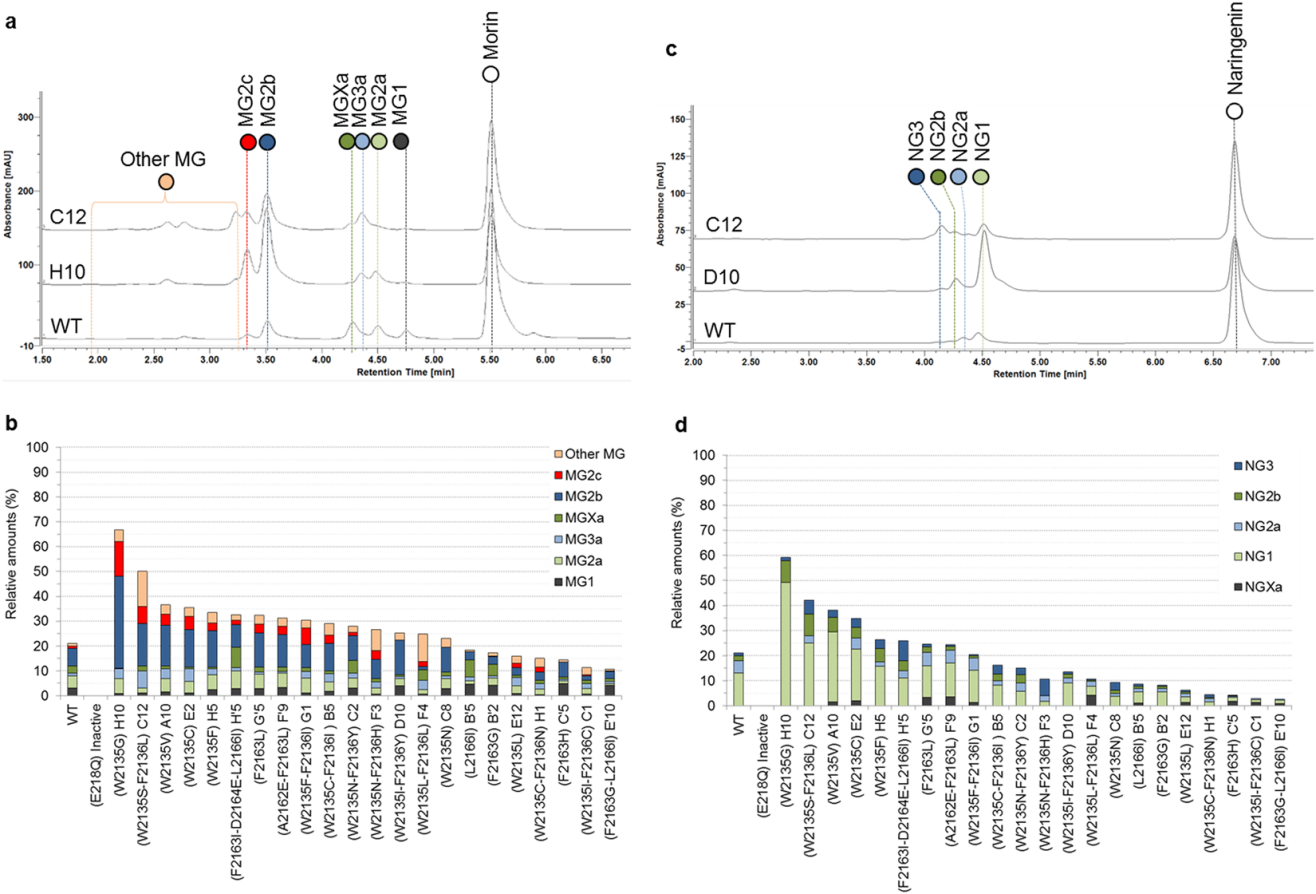
Morin and naringenin glucosylation profiles obtained with the 22 selected mutants. HPLC profiles of the best mutants for the production of each glucoside are reported for (**a**) morin and (**c**) naringenin. The products are named as ZGxn, where Z is M for morin and N for naringenin; G means Glucoside; x is the number of glucosyl units detected by mass spectrometry (X when couldn’t been defined); and n a letter to differentiate the products harboring the same number of glucosyl units. A table of MS data is also supplied as Supplementary Table S3. The relative amount of the glucosylation products obtained with the 23 selected mutants are given for (**b**) morin and (**d**) naringenin. Mutants are ranked relatively to the highest conversion rates. The products of low retention times produced in lower amounts are grouped under the denomination “Other MG” are explained in the Supplementary Table S3.

Morin and naringenin are less efficiently converted than luteolin or quercetin (Fig. 5). However, morin conversion was improved with 11 mutants, mutant C12 (W2135S-F2136L) and H10 (W2135G) being the most performing ones respectively with 47 and 67% conversion (20% for the wild-type enzyme). As seen before, substitution of the tryptophan residue clearly favor glucosylation. One mono-glucoside, two di-glucosides and one tri-glucoside were clearly identified on the LC/MS profile (Fig. 7a,b). The mutants C12, F4 and F3 synthesized higher amounts of morin glucosides referred to as “other MGs”, and which are likely glucosides of higher molar mass. The best mutant for morin glucosylation is mutant H10 (W2135G) that synthesized four times more MG2b glucoside than the wild-type (Fig. 7c).

Concerning naringenin, four mutants at position 2135 (H5 (W2135F), H10 (W2135G), A10 (W2135V) and D10 (W2135I-F2136Y) are also 3 to 4-fold improved compared to the wild-type. The highest conversion obtained with the double mutant D10 reached approximately 60%. Only seven reaction products were detected from naringenin acceptor reaction (Fig. 7c,d). One naringenin mono-glucoside, two di-glucosides and one tri-glucoside were clearly detected. Naringenin was poorly glucosylated by the wild-type enzyme (14%) and most of the converted naringenin was mono-glucosylated (NG1, 13%). Five mutants from library 2135–2136 exhibited an improved production of NG1, up to the 49% achieved with D10 mutant (W2135I-F2135Y). Two different mutants (C12 and F4) converted 10% of naringenin into a tri-glucoside form (versus 1% for the wild-type enzyme). In addition, it was previously reported that vicinal hydroxyl groups at positions 3′ and 4′ of ring B -as present in quercetin and luteolin- were required for flavonoid glucosylation by GH70 enzymes^24,25^. Our results indicate that the catechol ring is not a pre-requisite for glucosylation as both morin and naringenin that do not possess vicinal hydroxyl groups on ring B are converted with the wild-type enzyme and several mutants.

### Apigenin and Chrysin glucosylation

With only 2% conversion, apigenin and chrysin were both poorly recognized by the wild-type enzyme. The values obtained using the mutants never exceeded 5%.Compared to luteolin, apigenin and chrysin exhibit only one hydroxyl group at C4′ of ring B or no hydroxyl group in ring B, respectively. Apigenin displays an unbent structure, with a torsion angle below 30°, due to the presence of the C2,3-double bond^26,27^. The lack of flexibility of apigenin compared to naringenin, in which the C2,3 double bond is not present, may explain its limited reactivity. The very low conversion could also be attributed to the lower solubility of these flavonoids compared to the other ones tested^28^.

### Overall characteristics of the best mutants

**Figure 8:**
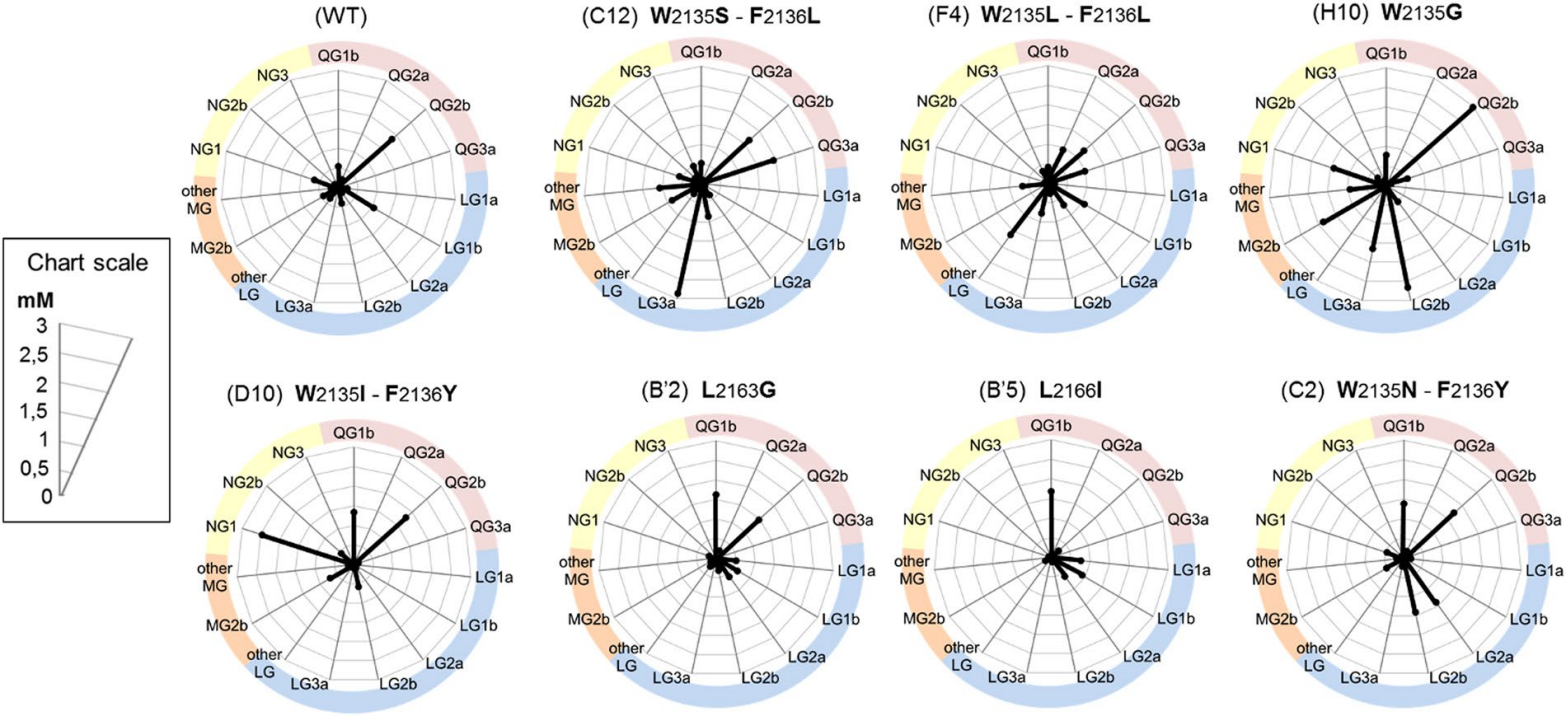
Production yield of the main flavonoid glucosides achieved by the 7 best mutants of the 2 libraries. All the reactions were conducted with 5 mM of aglycon flavonoid and 292 mM sucrose. The glucosides flavonoids are named according to the nomenclature established in the previous figures (Figs 6 and 7). Only flavonoid glucosides for which a mutant allow a production of at least 0,5 mM are represented. For the “Other MG” and “Other LG” only overall quantity of the different products grouped under that denomination is taken in account.

Figure 8 illustrates a compilation of the results obtained with the wild-type enzyme and the 7 most original variants. First, we can see that the α-1,2 branching sucrase is a good enzyme for flavonoid glucosylation, as the wild-type form can glucosylate four different flavonoids over the 6 tested. Notably, flavonoid structures that were never glucosylated by GH70 glucansucrases in previous studies, such as morin and naringenin, were obtained with this enzyme.

In GH70 family, the α-1,2 branching sucrases have naturally evolved to branch α-glucans, and we may assume that they are more adapted to glucosylate large molecules like flavonoids than the polymerases. Other flavonoid structures should be tested anyway with both branching sucrases and polymerases to confirm this hypothesis. In addition, Fig. 8 also highlights the strength of our approach. Indeed, our structurally guided and neutral variant library (for sucrose consumption and quercetin conversion) readily gave access to variants revealing higher product promiscuity (variant H10) or different specificities (C12, D10) compared to the wild-type enzyme (Fig. 8). The accessible diversity was easily enhanced from quite a limited number of variants, and variants reaching almost quantitative conversion could be generated.

## Conclusion

In this work, enzyme engineering efforts were focused on the catalytic pocket of the α-(1→2) branching sucrase. Four residues, not located in the vicinity of catalytic amino acids, were targeted and changed through saturation mutagenesis. From medium-size libraries, sucrose active mutants were rapidly isolated with a pH-based assay. Screening on quercetin glucosylation allowed further selection of a set of 22 variants which were more efficient than the wild-type enzyme. From this very small set of variants tested for their ability to glucosylate other structures of flavonoids, a high ratio of enzymes showing improved activities and leading to novel flavonoid glucosides was sorted out.

To go further and understand the role of the mutations onto mutant specificity, the isolation and structural characterization of the reaction products is now necessary. This will open the way to in-depth structure-function relationship studies that should help to further improve the variants and adapt them to the glucosylation of recalcitrant flavonoids such as apigenin and chrysin.

The mutants could also be further tested for the glucosylation of a larger panel of flavonoids. In addition, the same strategy of engineering could be applied, in the future, to other regions of the α-(1→2) branching sucrase (ΔN_123_-GBD-CD2) or to other GH70 enzymes, in order to extent the enzymatic platform and provide thereby a powerful tool for valorization of flavonoids in therapeutics, cosmetic or food-processing industries.

## Material and Methods

### Bacterial Strains, Plasmids, and Chemicals

*δn*123*-gbd-cd2* gene cloned in Gateway^®^ Nova pET-53-DEST™ DNA (Novagen) vector was used as DNA template for libraries construction^29^. *E. coli* TOP10 electrocompetent cells (Invitrogen, Carlsbad, USA) were used as host for the plasmid library and *E. coli* BL21 Star DE3 (Life Technologies) for gene expression. Fusion DNA-polymerase was purchased from Finnzymes (Espoo, Finland), and DpnI restriction enzyme from New England Biolabs (Beverly, MA, USA). Primers were synthesized by Eurogentec (Liège, Belgium). DNA extraction (QIASpin), gel extraction and purification (QIAQuick) kits were purchased from Qiagen (Chatsworth, CA). DNA sequencing was performed by GATC Biotech (Mulhouse, France). All positive selected clones for quercetin glucosylation were sequenced using forward primer 5′-CCAACGAACACGAATGGGC-3′ and the reverse one 5′-CTGTCATGATTGAATGCAAC-3′ surrounding the region encoding the motif V of catalytic domain that contains the targeted positions of the two mutant libraries (positions 2135–2136 and 2163–2166).

Apigenin, luteolin and naringenin were purchased from Carbosynth (Compton, Berkshire, UK); morin from Honeywell Riedel-de-Haën™ (Seelze, Germany); quercetin, chrysin, bromocresol purple (BCP), sucrose, lactose, glucose, glycerol, dimethylsulfoxide, 2-(N-morpholino)ethanesulfonic acid (MES), sodium acetate from Sigma–Aldrich (Saint-Louis, MO, USA); Ampicillin (Amp), lysozyme, and DNAse I from Euromedex (Souffelweyersheim, France); Durapore^®^ Membrane Filter 0.22 µm GV from Merck Millipore (Darmstadt, Deutschland).

### Molecular modeling and docking studies

The three-dimensional models of the α-(1→2) branching sucrase in complex with sucrose or mono-glucosylated quercetin were generated using as template the coordinates of the crystallographic structures of the α-(1→2) branching sucrase (ΔN_123_-GBD-CD2; PDB accession code: 3TTQ)^20^ and *L. reuteri* GTF-180 in complex with sucrose (PDB accession code: 3HZ3)^30^.

Molecules were initially manually docked in the active site of the enzymes. Four mono-glucosylated quercetin obtained from quercetin glucosylation at positions C3′ or C4′ of ring B and at positions C5 or C7 of ring A were constructed starting from the model of the α-(1→2) branching sucrase in complex with sucrose, in which the glucosyl moiety of sucrose was attached to the quercetin moiety, generated using Corina 3D converter (Molecular Networks, Erlangen, Germany). Glucosylation at position 3 of ring C from quercetin was left out due to major steric hindrance. Molecules were positioned using as template the complex of *Streptococcus mutans* GTF-SI in complex with acarbose inhibitor (PDB accession code: 3AIC)^31^. Mutations were introduced using the Biopolymer module of Insight II software package (Accelrys, Saint Louis, USA).

The conformation of the mutated residue side chain was optimized by manually selecting a low-energy conformation from a side-chain rotamer library. Steric clashes (van der Waals overlap) and non-bonded interaction energies (Coulombic and Lennard–Jones) were evaluated for the different side-chain conformations. All energy minimization calculations were performed using the CFF91 force field and steepest descent algorithm (10,000 iterations)^32^. Drawings were performed using PyMOL software^33^.

### Construction of the α-(1→2) branching sucrase libraries

The degenerated primers designed to achieve mutations at each position were: (i) 5′-GGTGGTGATGCT**NDTNDT**CAAGGTGGTTATCTGAAG-3′ and 5′-GATAACCACCTTG**AHNAHN**AGCATCACCACCACCATG-3′ for library 2135–2136; (ii) 5′-CCTGGTAATGCA**NDT**GATTTC**NDT**CTAGCCAACGACGTGG-3′ and 5′-GTCGTTGGCTAG**AHN**GAAATC**AHN**TGCATTACCAGGTTGACG-3′ for library 2163–2166. NDT and NHA codon indicates the bases which were used to obtain the replacement by the desired amino acids with N (A, T, C or G), D (not C) and H (not G). Inverse PCR amplifications were carried out using the high fidelity Phusion DNA polymerase (Thermo Scientific) according to the following protocol: Template DNA, 0.12 ng.µL^−1^; forward and reverse primers, 200 nM; dNTP, 200 µM each; Phusion DNA polymerase, 0.02 U/µL; in 50 µL of HF Buffer 1× (thermo Scientific). The PCR conditions were: 1 min start at 98 °C followed by 16 cycles of 98 °C −10s, 75 °C −15 s, 62 °C or 66 °C −30 s (degenerated oligos melting temperatures for library 2135–2136 and 2163–2166, respectively), 72 °C −10 min, and final extension of 10 min at 72 °C.

Amplified DNA was digested by DpnI endonuclease (20 U, 37 °C, 2 h) to eliminate the methylated parental template and loaded on a 0,8% agarose, Tris-acetate, EDTA (tris-base, 24,2 g.L^−1^; acetic acid, 5,7%; EDTA, 50 mM) gel for separation. The 8 kb bands, corresponding to pET53-*δn*_123_*-gbd-cd2* plasmids, were taken and purified using a Qiaquick Gel extraction kit (Qiagen^®^) and following the manufacturer recommendations. For each library, electrocompetent *E. coli* TOP10 were transformed with the amplified plasmids. The resulting clones were isolated by plating on LB agar (yeast extract, 5 g.L^−1^; Bacto Tryptone, 10 g.L^−1^; NaCl, 10 g.L^−1^; agar, 15 g.L^−1^) supplemented with 100 μg.mL^−1^ ampicillin. Clones were then scraped using 5 mL of physiological saline water per 22 cm square plate (Corning, USA), and plasmids containing the mutated *δn*_*123*_*-gbd-cd2* gene extracted. Purified DNA were quantified using a nanodrop (Thermo Scientific^™^) and freezed at −20 °C.

### α-(1→2) branching sucrase mutant libraries screening for sucrose consumption

The screening protocol was inspired from Champion *et al*.^34^ and is detailed in Supplementary Fig. S1 available. On the first day, library plasmid stocks were transformed into electrocompetent *E. coli* BL21 Star cells. Recombinant clones were first plated for six hours on a Durapore^®^ membrane, previously placed onto 22 cm square plates containing 200 mL of LB agar medium (200 mL) supplemented with ampicillin (100 mg.mL^−1^) to enable bacteria growth. The membranes were then transferred onto new square plates containing the expression solid medium, adapted from Vuillemin *et al*.^35^ as follows: solid agar ZYM5052 optimized with lactose, 0.5% w/v; glucose, 0.01%, w/v; glycerol, 0.5%, w/v; ampicillin, 100 mg.mL^−1^; adjusted at pH 6.1. The plates were then incubated at 23 °C during 2 days. On the fourth day, membranes were transferred onto new square plates containing the colorimetric screening medium: solid minimum M9 medium agar (Na_2_HPO_4_, 20 mM; KH_2_PO_4_, 40 mM; NH_4_Cl, 20 mM; NaCl 10 mM; CaCl_2_, 0.1 mM; pH 6.1) supplemented with lactose, 0.25% w/v; glycerol, 0.15%, w/v; thiamine, 5 µg.L^−1^; ampicillin, 100 mg.mL^−1^ and sucrose, 100 g.L^−1^. The medium was stained purple by addition of 50 mM 2-(*N*-morpholino)ethanesulfonic acid (MES) at pH 6.1, and bromocresol purple (BCP) indicator at 2.5 mg.L^−1^. The plates were incubated at 30 °C until clones expressing a sucrose active mutant turn yellow (12 to 24 h). Indeed, *E. coli* BL21 Star is derived from *E. coli* strain B and is naturally unable to use sucrose as substrate. Hence, only the transformants encoding active α-(1→2) branching sucrases can cleave sucrose substrate. The fructose and glucose molecules released can enter the glycolytic pathway, leading to acid production and concomitant pH decrease upon growth. The best pH compromise for growth and expression was obtained at pH 6.1. At this value, 40% of the enzyme activity was retained (Supplementary Fig. S1) and the active clone detection can be performed using bromocresol purple dye as pH indicator (BCP).

To validate this new screening method, a total of 16 clones (8 yellow and 8 purple-brown) were picked at random in a mix of clones expressing the wild-type α-(1→2) branching sucrase or an inactive mutant (E2248Q). Sequencing of the branching sucrase encoding genes confirmed that all the purple-green clones corresponded to the inactive mutants, whereas all the yellow ones corresponded to the wild-type enzyme encoding gene. For screened mutant libraries, positive clones (yellow) were picked into two 96-wells microplates containing LB medium (200 mL) supplemented with glycerol (8%, w/v) and ampicillin (100 mg.mL^−1^). After 24 h of growth at 30 °C, they were stored at −20 °C and −80 °C.

### Sucrose activity assay

All assays were performed at 30 °C in 50 mM sodium acetate, pH 5.75. Enzyme activity was determined by measuring the initial velocity of released fructose from 292 mM sucrose. Fructose concentration was determined using the dinitrosalycilic acid (DNS) method^36^. One unit of ΔN_123_-GBD-CD2 enzyme corresponds to the amount of enzyme that catalyzes the production of 1 μmol of fructose per min under the assay conditions.

### Semi-automated procedure for α-(1→2) branching sucrase mutant libraries screening with flavonoids

This screening method was adapted by combining the previously optimized methods described in^16,35^. Storage microplates of the single mutants expressing clones were thawed and used to inoculate 96-well microplates containing, in each well, 150 μL of LB medium supplemented with ampicillin (100 μg/mL). After 24 h growth at 30 °C under agitation (200 rpm), plates were duplicated into 96-Deep Well plates containing, in each well, 1 mL of optimized autoinducible ZYM5052 medium (containing 0.75% α-lactose, 0.05% glucose, 1.5% glycerol) supplemented with ampicillin (100 μg/mL). Cultures were then grown for 32 h at 23 °C under agitation (200 rpm). The plates were centrifuged (20 min, 3000 g, 4 °C), cell pellets were resuspended in 300 μL of 20 mM sodium acetate buffer at pH 5.75, supplemented with lysozyme (0.5 mg/mL) and DNase I (2.5 U/ml) and freezed at −80 °C for 8 to 12 h. After thawing at room temperature, raw extracts were centrifuged (30 min, 2500 g, 4 °C). Supernatants, corresponding to the soluble enzymatic fraction, were then transferred in 96-well microplates and stored on ice. Flavonoid glucosylation reactions were carried out in 300 µL final volume (96-Deep well microplates) in sodium acetate buffer 20 mM pH 5.75 with 292 mM sucrose, 5 mM flavonoids and 10% DMSO. Reactions were incubated 20 h at 30 °C under agitation (infors, 700 rpm) and stopped by heating at 95 °C for 5 min. Sucrose consumption and luteolin conversion rate were then evaluated for each mutant.

### Analytical methods

Sucrose consumption was monitored by high performance liquid chromatography analysis, as previously described in Malbert *et al*.^16^, using a Dionex P 680 series pump equipped with an autosampler HTC PAL and a Shodex RI 101 series refractometer, and a Biorad HPLC Carbohydrate Analysis column (HPX-87K column (300 mm × 7.8 mm)) maintained at 65 °C. Ultrapure water was used as eluent with a flow rate of 0.6 mL/min. Data acquisition and processing were performed using Chromeleon 6.80 data systems. Reaction samples were diluted in 4 volumes of ultrapure water and centrifuged (2 min, 10000 g) to remove insoluble compounds. The percentages of sucrose consumption (%Sc) were determined as follows:

1 $$ \% Sc=\frac{{[Sucrose]}_{T0}-\,{[Sucrose]}_{Tf}}{{[Sucrose]}_{T0}}\times 100$$

where [Sucrose]_T0_ corresponds to the initial concentration of sucrose in mol/kg, and [Sucrose]_Tf_ is the concentration of sucrose in mol/kg at the end of the reaction.

Flavonoids and their glucosylated forms were analyzed by LC-MS analysis, as previously described in Malbert *et al*.^16^, using a DIONEX Ultima 3000 series chromatograph equipped with a Dionex UVD 340 UV/vis detector and coupled with a simple-quadrupole mass spectrometer (MSQ Plus, Thermo Scientific). HPLC analyses were performed using a reversed phase analytical column (Prontosil Eurobond, 3 μm, 53 mm × 4.6 mm; Bischoff Chromatography, Germany) maintained at 40 °C. Data acquisition and processing were performed using MSQ Plus 2.0 SP1 and Chromeleon 6.80 data systems. Samples were analyzed using the following gradient starting from 15% B to 25% B over 3 min and from 25% B to 50% B over 3.5 min (A: ultrapure water with 0.1% formic acid; B: acetonitrile with 0.1% formic acid) at a flow rate of 0.5 mL/min. Detection of flavonoid compounds was monitored at 255 and 340 nm. The samples were diluted with 15 volumes of DMSO to solubilize flavonoids. The amount of converted flavonoids were calculated by summing the moles of glucosylated products identified from HPLC analysis after checking that the UV response factors of the various products were similar. Flavonoids conversion were determined as the ratio of consumed flavonoid versus theoretical concentration at initial time (taking into account both the solubilized and the insoluble fraction).

The HPLC system was coupled to the mass spectrometer equipped with electrospray ionization (ESI) ion source according to the following parameters: desolvation gas temperature 450 °C, electrospray capillary voltage 5 kV in negative mode, source block temperature 150 °C and cone voltage 50 V and 110 V. Nitrogen was used as drying gas and nebulizing gas at flow rates approximately 80 L/h. The mass spectrometer scanned from m/z 100 to 1,500.

## Electronic supplementary material

Supplementary Figures and Tables

## Data Availability

All data generated or analyzed during this study are included in this published article (and its Supplementary Information files). The datasets generated and/or analyzed during the current study are available from the corresponding author on reasonable request.
