# Supplementary material for: Engineering a branching sucrase for flavonoid glucoside diversification
Source: Sci Rep. 2018 Oct 11;8:15153. doi: 10.1038/s41598-018-33394-y (PMC6181985; doi:10.1038/s41598-018-33394-y)
Supplement: Supplementary file 1 — Supplementary Figures and Tables [file 41598_2018_33394_MOESM1_ESM.pdf]

Engineering a branching sucrase for flavonoid glucoside diversification

Yannick Malbert[1], Claire Moulis[1], Yoann Brison[1], Sandrine Morel[1], Isabelle André[1] & Magali Remaud-Simeon[1*]

[1] Laboratoire d'Ingénierie des Systèmes Biologiques et Procédés, LISBP, Université de Toulouse, CNRS, INRA, INSA, Toulouse, France. 135, avenue de Rangueil, F-31077 Toulouse cedex 04, France
* Correspondence and requests for materials should be addressed to M.R-S. (email: remaud@insa-toulouse.fr)

Supplementary Figures and Tables

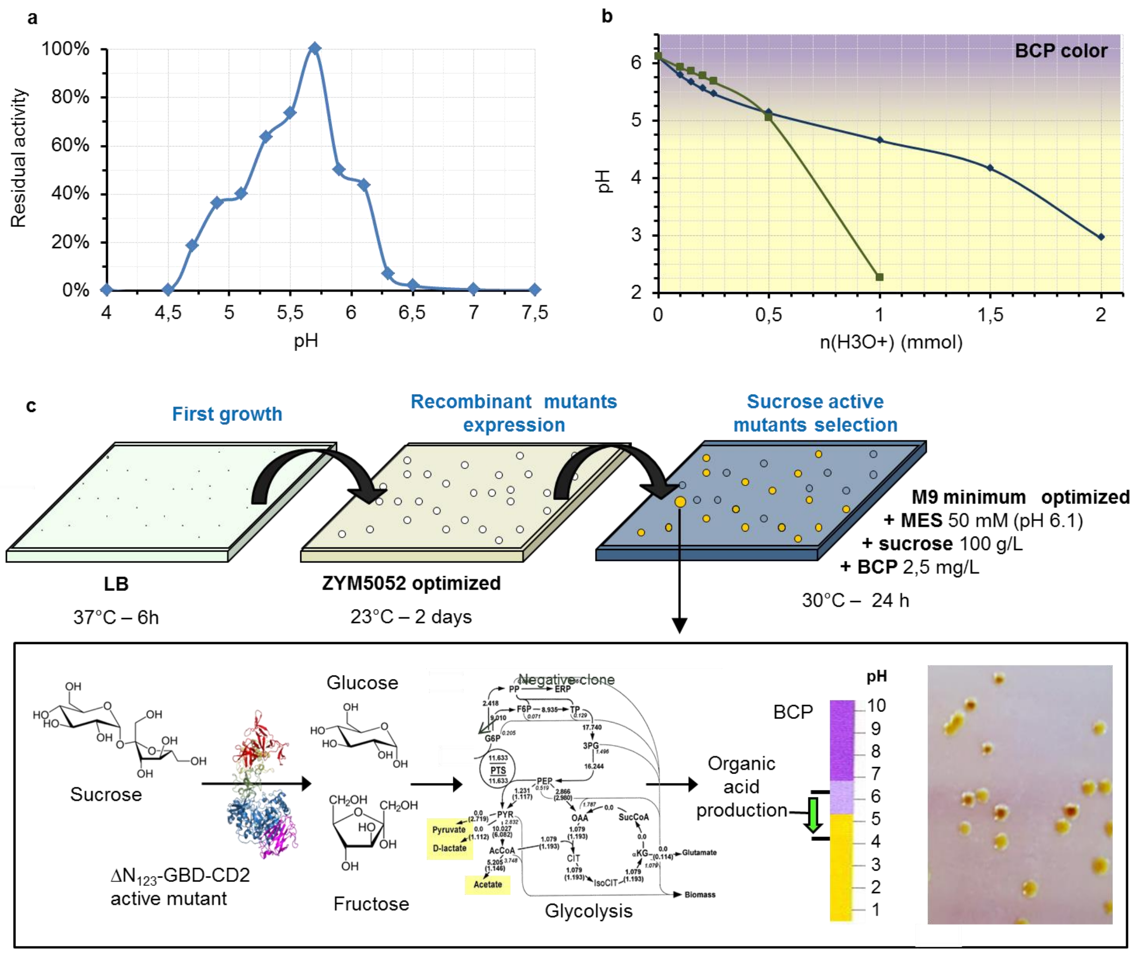

Figure S1. Solid-state pH-based colorimetric screening method. (a) Residual activity of the α-(1→2) branching sucrase as a function of pH. Residual activities were calculated by comparison to activities determined before enzyme storage at particular pH. (b) pH and BCP color variation versus H3O+ addition. Blue and green curves correspond respectively to sodium acetate and MES buffer at 50 mM and initial pH value of 6.1.(c) Screening method principle and validation with a mix of clones expressing wild-type or inactive α-(1→2) branching sucrase (mutant E2248Q). Yellow colonies correspond to clones active on sucrose (positive clones), whereas purple-green ones are inactive ones (negative clones).

a

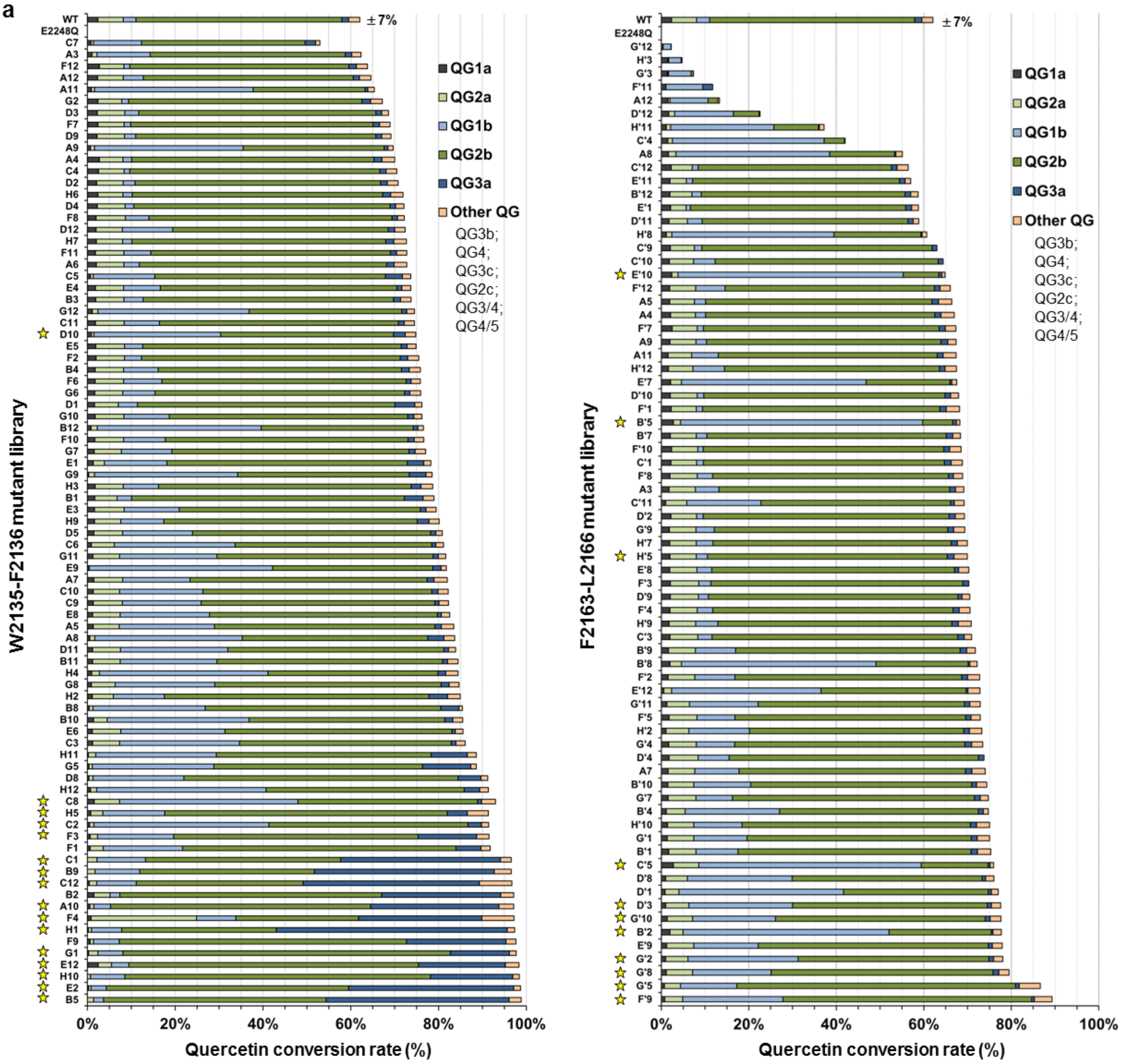
W2135-F2136 mutant library
WT
E2248Q
C7
A3
F12
A12
A11
G2
D3
F7
D9
A9
A4
C4
D2
H6
D4
F8
D12
H7
F11
A6
C5
E4
B3
G12
C11
D10
E5
F2
B4
F6
G6
D1
G10
B12
F10
G7
E1
G9
H3
B1
E3
H9
D5
C6
G11
E9
A7
C10
C9
E8
A5
A8
D11
B11
H4
G8
H2
B8
B10
E6
C3
H11
G5
D8
H12
C8
H5
C2
F3
F1
C1
B9
C12
B2
A10
F4
H1
F9
G1
E12
H10
E2
B5
±7%
QG1a
QG2a
QG1b
QG2b
QG3a
Other QG
QG3b;
QG4;
QG3c;
QG2c;
QG3/4;
QG4/5
0%
20%
40%
60%
80%
100%
Quercetin conversion rate (%)
F2163-L2166 mutant library
WT
E2248Q
G'12
H'3
G'3
F'11
A12
D'12
H'11
C'4
A8
C'12
E'11
B'12
E'1
D'11
H'8
C'9
C'10
E'10
F'12
A5
A4
F'7
A9
A11
H'12
E'7
D'10
F'1
B'5
B'7
F'10
C'1
F'8
A3
C'11
D'2
G'9
H'7
H'5
E'8
F'3
D'9
F'4
H'9
C'3
B'9
B'8
F'2
E'12
G'11
F'5
H'2
G'4
D'4
A7
B'10
G'7
B'4
H'10
G'1
B'1
C'5
D'8
D'1
D'3
G'10
B'2
E'9
G'2
G'8
G'5
F'9
±7%
QG1a
QG2a
QG1b
QG2b
QG3a
Other QG
QG3b;
QG4;
QG3c;
QG2c;
QG3/4;
QG4/5
0%
20%
40%
60%
80%
100%
Quercetin conversion rate (%)

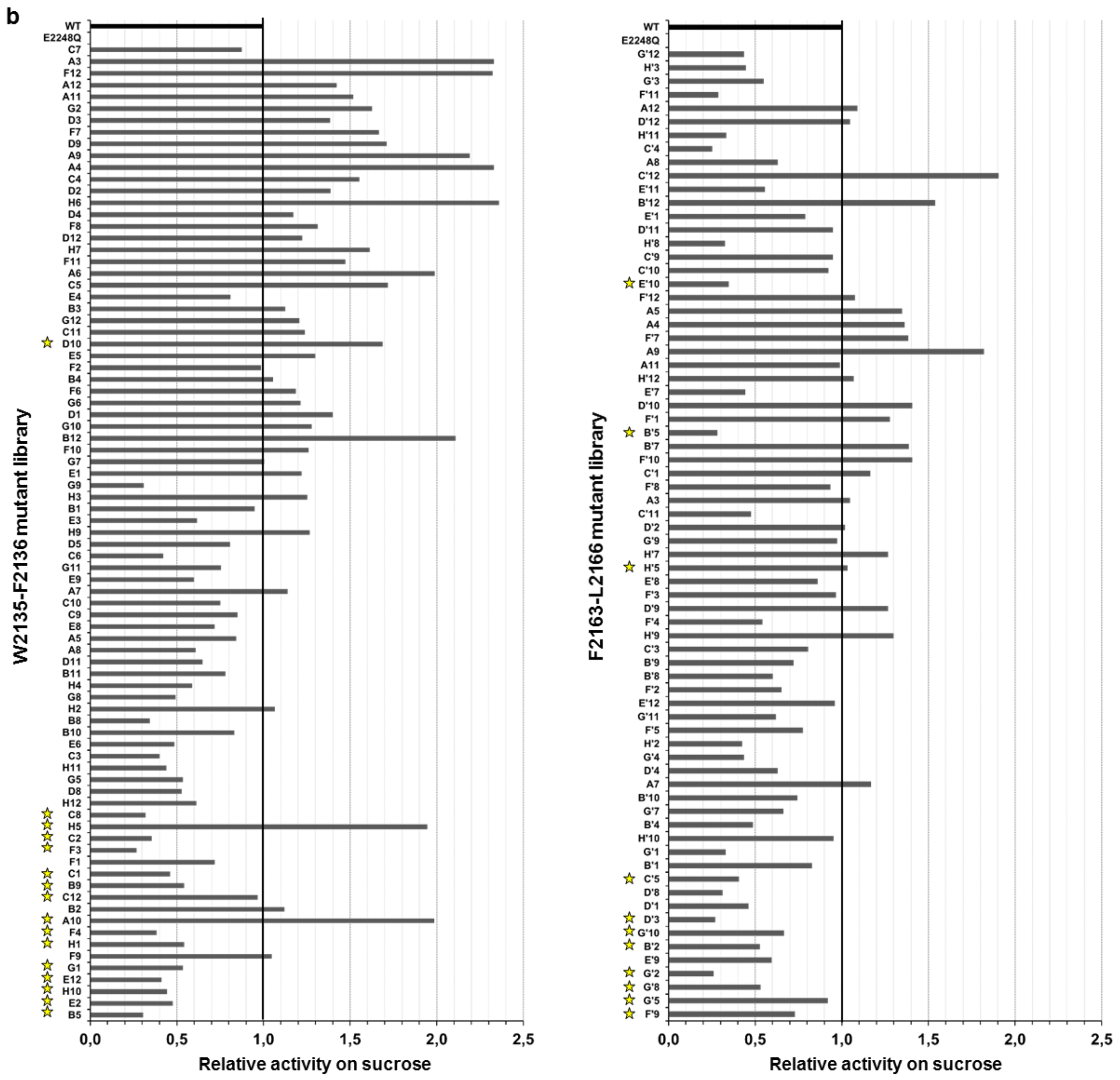

Figure S2. Results of the secondary screening of the α-(1→2) Bsr mutant libraries. for their capacity to glucosylate quercetin. (a) Quercetin conversion rates for mutants belonging to library W2135-F2136 and F2163-L2166. Selected mutants are highlighted with a ★. The proportions of each reaction product were calculated according to the formula given in the method section. Standard deviations are given for the WT (calculated on 3 replicates). (b) Relative activities of mutants on sucrose in absence of flavonoid, compared to the parental enzyme. The activity were measured by DNS assays as described in the method section.

Table S3. HPLC-MS data of the different glucosylation products obtained for the reactions of the 22 mutants of the α-(1→2) branching sucrase with quercetin, luteolin, morin and naringenin. The numbers of glucosyl units are calculated according to the detected m/z of the compounds.

Group / Name		Retention time (min)	m/z *	Number of glucosyl units *
**Quercetin**		6.0	301	0
QG1a		5.1	463	1
QG2a		4.9	625	2
QG1b		4.7	463	1
QG2b		4.5	625	2
QG3a		4.4	787	3
**Other QG**	QG3b	4.1	787	3
	QG4	3.8	949	4
	QG3c	3.5	787	3
	QG2c	3.3	625	2
	QG3/4	2.9	787 ; 949	3 to 4
	QG4/5	2.4	949 ; 1112	4 to 5
**Luteolin**		5.8	285	0
LG1a		4.7	447	1
LG2a		4.5	609	2
LG1b		4.4	447	1
LG2b		4.2	609	2
LG3a		4.1	771	3
**Other LG**	LG2c	3.9	609	2
	LG3b	3.6	771	3
	LG4	3.3	934	4
	LG2d	3.2	609	2
	LG3c	2.7	771	3
	LG3/4	2.6	771 ; 933	3 to 4
	LG4/5	2.1	933 ; 1094	4 to 5
**Morin**		5.5	301	0
	MG1	4.7	464	1
	MG2a	4.5	626	2
	MG3	4.3	788	3
	MGXa	4.2	n.a.	n.a.
	MG2b	3.5	626	2
	MG2c	3.3	626	2
**Other MG**	MGXb	3.2	n.a.	n.a.
	MGXc	2.8	n.a.	n.a.
	MGXd	2.6	n.a.	n.a.
	MGXe	2.4	n.a.	n.a.
	MGXf	2.2	n.a.	n.a.
**Naringenin**		6.7	271	0
NGXa		5.3	n.a.	n.a.
NG1		4.5	432	1
NG2a		4.4	594	2
NG2b		4.3	594	2
NG3		4.2	756	3

* n.a. (not assigned) is given when no significant m/z, in the MS spectrum, could be determined.
